# Engineering Multi‐Scale Organization for Biotic and Organic Abiotic Electroactive Systems

**DOI:** 10.1002/advs.202205381

**Published:** 2023-01-20

**Authors:** Ze‐Fan Yao, Emil Lundqvist, Yuyao Kuang, Herdeline Ann M. Ardoña

**Affiliations:** ^1^ Department of Chemical and Biomolecular Engineering Samueli School of Engineering University of California Irvine CA 92697 USA; ^2^ Department of Chemistry School of Physical Sciences University of California Irvine CA 92697 USA; ^3^ Department of Biomedical Engineering Samueli School of Engineering University of California Irvine CA 92697 USA; ^4^ Sue & Bill Gross Stem Cell Research Center University of California Irvine CA 92697 USA

**Keywords:** bioelectronics, conjugated polymers, electroactive polymers, patterning, self‐assembly

## Abstract

Multi‐scale organization of molecular and living components is one of the most critical parameters that regulate charge transport in electroactive systems—whether abiotic, biotic, or hybrid interfaces. In this article, an overview of the current state‐of‐the‐art for controlling molecular order, nanoscale assembly, microstructure domains, and macroscale architectures of electroactive organic interfaces used for biomedical applications is provided. Discussed herein are the leading strategies and challenges to date for engineering the multi‐scale organization of electroactive organic materials, including biomolecule‐based materials, synthetic conjugated molecules, polymers, and their biohybrid analogs. Importantly, this review provides a unique discussion on how the dependence of conduction phenomena on structural organization is observed for electroactive organic materials, as well as for their living counterparts in electrogenic tissues and biotic‐abiotic interfaces. Expansion of fabrication capabilities that enable higher resolution and throughput for the engineering of ordered, patterned, and architecture electroactive systems will significantly impact the future of bioelectronic technologies for medical devices, bioinspired harvesting platforms, and in vitro models of electroactive tissues. In summary, this article presents how ordering at multiple scales is important for modulating transport in both the electroactive organic, abiotic, and living components of bioelectronic systems.

## Graphical abstract

Multi‐scale organization of electroactive organic materials is crucial for regulating charge transport in bioelectronic systems. This review summarizes approaches to control the organization of electroactive organic materials and living systems, ranging from molecular order, nanoscale assembly, microstructure domains, and macroscale architectures. Controlling the multi‐scale organization of such systems will advance bioelectronic technologies for medical devices, bioinspired harvesting platforms, and in vitro models of electroactive tissues.

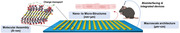

## 1 Introduction

Electroactive organic materials, including synthetic polymers/ biomolecule‐based materials and their related devices, have emerged as key functional components of bioelectronic technologies over the past decades.^[^ ^1^, ^2^, ^3^, ^4^, ^5^, ^6^, ^7^ ^]^ The conduction mechanisms of these organic materials can involve through‐bond or through‐space ionic and electronic contributions, unlike their inorganic counterparts that often involve delocalized electron transport.^[^ ^8^, ^9^, ^10^ ^]^ Efficient electronic transport is required to achieve high‐performance devices, while facilitation of ion transport within polymeric or biomolecular networks allows for easy integration with electrolytic biological environments.^[^ ^8^, ^11^ ^]^ Both mechanisms for electroactive organic materials are dependent on molecular and supramolecular order, and even on higher‐scale organization spanning multiple dimensions. For example, ordered supramolecular nanostructures of conducting polymers can accelerate the mixed ionic‐electronic transport simultaneously.^[^ ^12^, ^13^ ^]^ As a result, this mixed nature of species that contribute to their order‐ and structure‐dependent charge transport make organic materials as attractive electroactive components of emerging bioelectronic technologies such as for biosensors,^[^ ^11^, ^14^, ^15^, ^16^ ^]^ tissue engineering,^[^ ^3^, ^17^, ^18^ ^]^ biologically relevant devices,^[^ ^19^, ^20^ ^]^ and other healthcare applications.^[^ ^21^, ^22^, ^23^, ^24^ ^]^ Thus, precision engineering of organization across multiple length scales for these materials—from molecular assembly to macroscopic architecture—is critical for pushing the boundaries of their functionalities and performance as electroactive components for the future of biomedical technologies such as in flexible electronics, wearable smart devices, and personalized medicine.^[^ ^25^, ^26^, ^27^ ^]^

**Figure 1:**
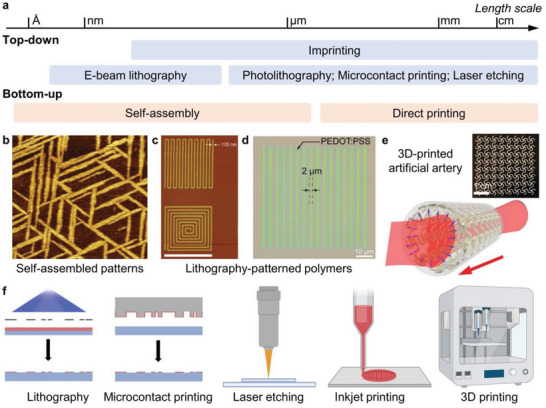
Currently available fabrication strategies for controlling the organization of electroactive organic materials across multiple dimensions. a) Fabrication approaches discussed herein and their corresponding length scales. b) Self‐assembled patterns of a peptide on MoS_2_ surface. Reproduced with permission.^[^ ^36^ ^]^ Copyright 2018, American Association for the Advancement of Science (AAAS). c) Nanoscale patterns of a block polymer formed by E‐beam lithography. Reproduced with permission.^[^ ^37^ ^]^ Copyright 2008, American Chemical Society (ACS). d) 2‐µm line patterns of PEDOT:PSS by direct optical lithography. Reproduced with permission.^[^ ^38^ ^]^ Copyright 2021, AAAS. e) 3D‐printed artificial artery Reproduced with permission.^[^ ^39^ ^]^ Copyright 2020, Wiley‐VCH Verlag GmbH & Co. f) Schematic diagram of representative patterning techniques. A portion of this figure was created with BioRender.com.

Advancements in inorganic, particularly silicon‐based, devices offer a great foundation for fabrication technologies that enable the integration and miniaturization of electronic devices with high resolution and accuracy. Several of these patterning techniques used in inorganic semiconductors or integrated microdevices are translatable for generating ordered complex structures, with up to nanoscale resolution, using electroactive organic materials.^[^ ^28^, ^29^, ^30^, ^31^, ^32^, ^33^ ^]^ However, the weak van der Waals interactions in polymeric materials or biomolecular assemblies make it challenging to fine tune the formation of ordered structures at different length scales using fabrication techniques that have been historically optimized for silicon‐based devices such as photolithography. Additional design and fabrication parameters also need to be considered for engineering biohybrid interfaces using the parting order via a bottom‐up or top‐down strategy for electroactive organic materials at different length scales (**Figure** **1**).^[^ ^34^, ^35^ ^]^

**Figure 2:**
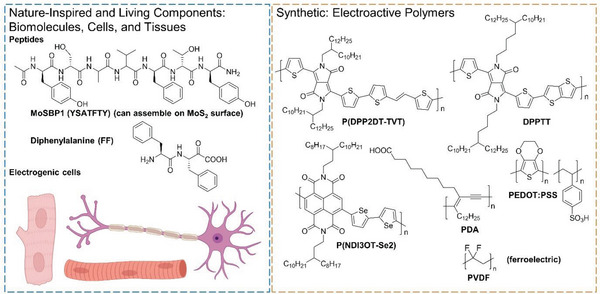
Material scope and chemical structures of representative bioinspired and synthetic electroactive organic materials discussed in this review. A portion of this figure was created with BioRender.com.

In this review, we highlight the current progress and challenges in engineering the multi‐scale organization of electroactive organic materials—from synthetic polymers, to bioinspired systems, to those that are integrated or interfaced with living components (**Figure** **2**). Here, we provide a thorough discussion of the state‐of‐the‐art on organizational approaches for these electroactive systems, as well as current challenges that should be overcome to continue the emergence of organic materials for bioelectronic applications. In particular, this article discusses electroactive systems from the engineering and biology perspectives, as we present how current fabrication approaches can impart structural organization and consequently influence conduction mechanisms in organic materials (abiotic), analogous living (biotic) electroactive systems, and at the interface of these two regimes.

## 2 Strategies for Implementing Molecular, 2D, and 3D Organization in Electroactive Organic Materials

Here, we summarize the strategies and approaches for implementing molecular, 2D, and 3D organization in electroactive organic materials (Figure 1 and **Table** **1**), highlighting their potential use in biological studies. These techniques that control the structural organization of electroactive organic materials are also known to consequently enable precise tunability of material properties. Several efforts have been devoted to controlling material organization at multiple length scales, because the structure−function relationship of electroactive organic materials is a multi‐scale phenomenon that can be attributed to hierarchical organization from molecular structures, nanoscale assembly, microstructural domains, and macroscale architectures. Although there are previous reports focused on how multi‐scale structures could affect the properties of electroactive organic materials, examples of which will be described in more detail in the next sections, it is expected that a more holistic and predictable structure−function relationship from programmable molecular structures, hierarchical organization, to biointerfacing properties will emerge in future reports.

**Table 1:** Strategy for controlling the structural organization of organic electroactive materials at multiple length scales

Strategy	Advantages	Disadvantages
Self‐assembly	Tunable by molecular design Formation of nanostructures Tunable morphology	Additional material synthesis
Lithography‐based methods	Large‐scale fabrication High resolution Easy pattern design	Difficult to directly pattern organic molecular materials Requires crosslinkers and prepatterned masks
Microcontact printing	Layer‐by‐layer Direct patterning	Requires pre‐prepared stamps Difficult for large‐scale fabrication
Laser etching	No need for a mask or treatment with hazardous chemicals Easy pattern design	Point‐by‐point exposure Time‐consuming
Inkjet printing	Drop‐by‐drop High throughput Tunable resolution Easy pattern design	Requires material solubility in ink Difficult to achieve resolution < micrometer Possibility of ink clogging in nozzles Challenges for continuity between patterns Limited 2D patterns
3D printing	Capable of line‐by‐line fabrication High throughput External solidification cues Easy pattern design	Requires material solubility in ink and viscosity

### 2.1 Ordering at the Molecular Level: Self‐Assembly Principles

**Figure 3:**
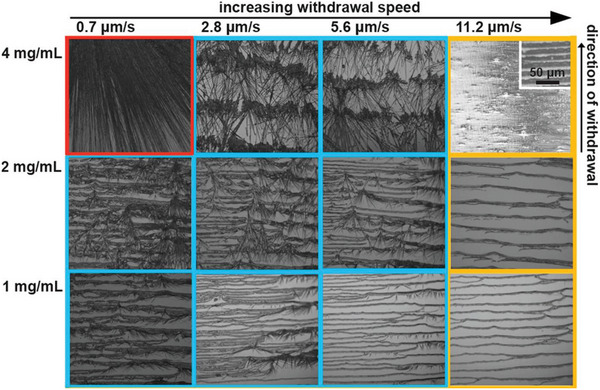
Ordered micro‐ and nano‐ structures of diphenylalanine (FF) through self‐assembly and dip‐coating process. Optical microscopy images of FF assemblies formed from dip‐coating process with different withdrawing speeds and initial concentrations. The FF aqueous solution contained 0.027 wt% NH_4_OH while the local nitrogen flow was 1 L min^−1^. Reproduced with permission.^[^ ^64^ ^]^ Copyright 2022, ACS.

Charge‐transporting organic assemblies that rely on self‐assembly present the advantage of having structures that can be modulated by intermolecular interactions via molecular design.^[^ ^40^, ^41^, ^42^, ^43^, ^44^ ^]^ Peptides and proteins, along with their functionalized analogs, represent a class of biomolecules that can form ordered supramolecular structures^[^ ^45^, ^46^, ^47^, ^48^, ^49^ ^]^ and also exhibit conductive or electroactive properties in solid‐state or in gels.^[^ ^50^, ^51^, ^52^, ^53^, ^54^, ^55^ ^]^ Among small biomolecules, diphenylalanine (FF) and its derivatives have been one of the most well‐established self‐assembling peptide to demonstrate semiconducting and ferroelectric properties.^[^ ^50^, ^56^, ^57^, ^58^, ^59^ ^]^ For example, Gazit and coworkers reported that FF can self‐assembled into ordered nanotubes.^[^ ^60^ ^]^ Furthermore, Gazit, Rishpon, and coworkers demonstrated that modification layer of assembled FF nanotubes on the graphite or gold electrodes can enhance the electrochemical activity and sensing selectivity.^[^ ^61^, ^62^ ^]^ Different substituent groups are introduced with FF, providing more interaction sites to tune the self‐assembly, such as 9‐fluorenylmethoxy‐carbonyl (Fmoc) and thiol group.^[^ ^56^, ^63^ ^]^ Typically, strong hydrogen bonding among peptide residues and ordered *π*–*π* stacking between benzene rings will benefit the self‐assembly into larger and ordered nanostructures of FF.^[^ ^56^ ^]^ Lee et al. investigated crystal structures, photophysics, and semiconducting properties of a cyclic diphenylalanine (cyclo‐FF). Nanowire device of cyclo‐FF clearly showed linear current−voltage relationship and temperature‐enhanced conductivity, indicating semiconductive charge transport.^[^ ^57^ ^]^ Liu et al. reported ordered micro‐ and nanostructures of FF by controlled self‐assembly and dip‐coating process (**Figure** **3**).^[^ ^64^ ^]^ They adopted diphenylalanine in aqueous ammonia solutions to promote peptide molecules forming aggregates during the dip‐coating process and solvent evaporation. Several deposition process parameters were systematically investigated to produce controlled morphologies, including dip‐coating withdraw speed, external nitrogen flow, peptide, and ammonia concentrations. By tuning these deposition process parameters, FF could form different fiber morphologies varying from long and straight microfibers and shorter curved nanofibers to uniform individual stripes with regular widths and spacings. Therefore, this work demonstrates a simple self‐assembly approach to build ordered 2D peptide assembly structures with well‐controlled morphologies.

**Figure 4:**
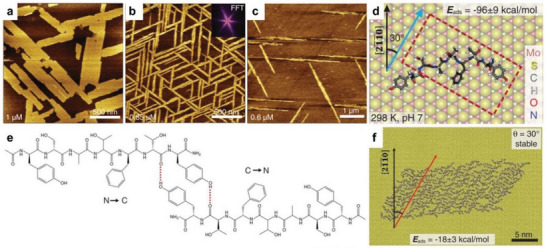
Ordered nanostructures of peptides self‐assembled on inorganic surfaces. a–c) AFM images of the self‐assembled nanostructures of a peptide sequence (YSATFTY) on MoS_2_ (0001), formed under various concentrations (1, 0.85, and 0.6 µm). d) Favorable binding conformation of a single unit of YSATFTY on MoS_2_ (0001) from MD simulations. e) Proposed dimer arrangement stabilized by intermolecular hydrogen bonds. f) Representative MD snapshot of the stable assemblies of YSATFTY on MoS_2_ (0001). Reproduced with permission.^[^ ^36^ ^]^ Copyright 2018, AAAS.

In other cases, interactions between biomolecules such as peptides are among those that have been utilized as templating group that drives the ordering of electroactive *π*‐conjugated units.^[^ ^52^, ^53^, ^65^ ^]^ Although the self‐assembly of these biomolecules is well established in solution phase, the production of ordered assemblies and specific patterns on device surfaces remains challenging. Approaches to date have been able to utilize features on nanostructured surfaces to impart assembly of biomolecules. For example, Huang, Yoreo, and coworkers investigated the self‐assembly of a peptide sequence (Tyr‐Ser‐Ala‐Thr‐Phe‐Thr‐Tyr, YSATFTY) on MoS_2_ (0001) surface and demonstrated the 2D ordered patterns (**Figure** **4**a–c) by avoiding the nucleation barrier.^[^ ^36^ ^]^ They utilized in situ AFM characterization to systematically study the dynamic process of molecular assembly, nucleation, and further growth of YSATFTY on MoS_2_ surface, supported by molecular dynamics (MD) simulations (Figure 4d). They found that the peptide molecules of YSATFTY could assemble one row at a time on MoS_2_ surface. The ordered assembled nanostructures result from the intermolecular hydrogen bonds and *π*−*π* stacking interactions between phenyl rings and preferential growth alignment on MoS_2_ (0001) surface (Figure 4e). MD simulations provided possible molecular stacking mode of YSATFTY on MoS_2_ (0001) surface with relative energies and supported the stable stacking alignment along specific crystal directions (Figure 4f). Therefore, nuclei of YSATFTY on MoS_2_ surface were ordered and formed without a free energy barrier, promoting the 2D growth habit. This work provides an exemplar demonstration of utilizing a self‐assembly guide for controlling molecular packing order, which can be used to achieve molecular‐level order in electroactive devices. On the other hand, more work is expected on how peptides form 2D assemblies on inorganic or bio‐based materials, especially the molecular structure effect on assembly and functions.^[^ ^66^ ^]^

**Figure 5:**
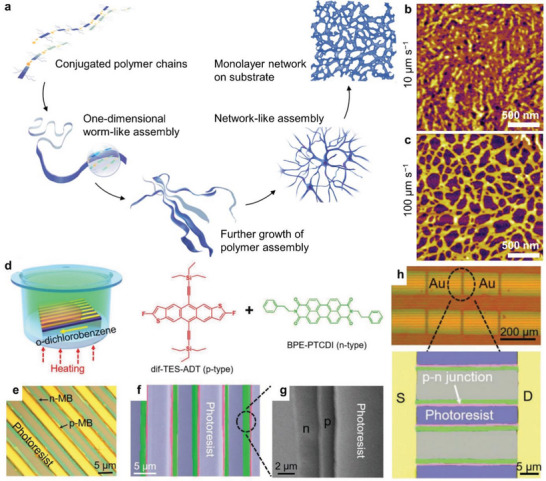
Ordered nanostructures of conjugated molecules and polymers through self‐assembly. a) Multi‐level self‐assembly process of a F_4_BDOPV‐based conjugated polymer. AFM height images of deposited polymer film and network monolayer on SiO_2_/Si substrates at different dip‐coating speeds of b) 10 and c) 100 µm s^−1^, respectively. Reproduced with permission.^[^ ^70^ ^]^ Copyright 2019, WILEY‐VCH Verlag GmbH & Co. d) Schematic diagram of the confined growth process of p‐n junction arrays of two semiconducting conjugated molecules. e) Optical microscopy image of the p–n junction arrays. f,g) Colored and enlarged scanning electron microscopy images of the p–n junction arrays. h) Optical microscopy image of the field‐effect transistors based on p–n junction arrays. Reproduced with permission.^[^ ^42^ ^]^ Copyright 2018, WILEY‐VCH Verlag GmbH & Co.

**Figure 6:**
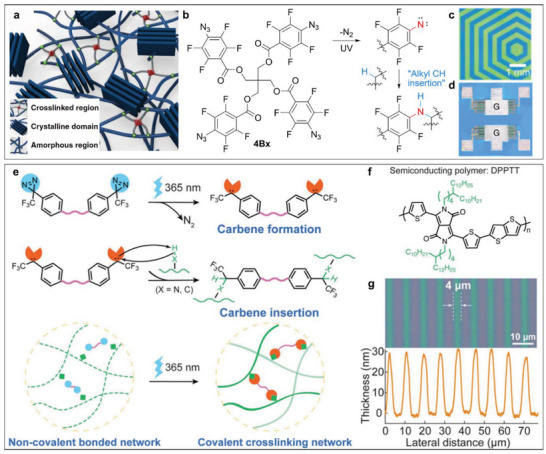
Crosslinking‐inspired direct lithography‐base patterning of conjugated polymers. a) Schematic illustration of crosslinked polymer chains using the 4‐arm crosslinker of 4Bx. b) Chemical structure and mechanism of the 4‐arm crosslinker of 4Bx. Optical image of c) the polymer patterns and d) a field‐effect transistor of P(DPP2DT‐TVT). Reproduced under the Creative Commons Attribution 4.0 license.^[^ ^80^ ^]^ Copyright 2020, The Authors. Published by Springer Nature. e) Mechanism of direct optical lithography for semiconducting and dielectric polymers based on UV‐triggered carbene insertion crosslinking. f) Chemical structure of a conjugated polymer of DPPTT. g) Optical image of 4‐µm line patterns of DPPTT and corresponding height profile by direct optical lithography. Reproduced with permission.^[^ ^38^ ^]^ Copyright 2021, AAAS.

Self‐assembly strategies have also been used to control the multi‐scale structural order of conjugated molecules and polymers toward specific functions.^[^ ^19^, ^20^, ^67^, ^68^, ^69^ ^]^ Pei and coworkers demonstrated the large‐scale fabrication of uniform 2D monolayer networks using a typical n‐type conjugated polymer (F_4_BDOPV‐2T) via a multi‐level self‐assembly strategy (**Figure** **5**).^[^ ^70^ ^]^ By tuning solvents, F_4_BDOPV‐2T formed 1D worm‐like assemblies in the solution due to the strong *π*–*π* interactions, which was confirmed via small angle neutron scattering (SANS) of the polymer solutions and microscopy images of the freeze‐dried samples. These 1D worm‐like assemblies could further grow to network‐like assemblies in solution. During the film‐formation process, uniform 2D monolayer networks could be deposited on substrates up to 4‐inch wafers. AFM height images (Figure 5b) indicate that F_4_BDOPV‐2T thin films from the chloroform solution were continuous multilayers when the dip‐coating rate was 10 µm s^−1^. As the dip‐coating rate increased to ≈20–500 µm s^−1^, uniform monolayer networks could be observed with similar thicknesses around 4 nm (Figure 5b). Absorption spectra and thin‐film X‐ray scattering also proved the polymer monolayer characteristic of the processed material. The resulting ordered monolayer networks of F_4_BDOPV‐2T exhibited high electron transport mobilities of up to 1.88 cm^2^ V^−1^ s^−1^ in field‐effect transistors. Briefly, this work provides an effective self‐assembly strategy to prepare large‐scale and uniform 2D polymer monolayers with specific structural order. By tuning polymer aggregation with temperature, Pei and coworkers further obtained ordered solid‐state microstructures in solution‐processed thin films of conjugated polymers.^[^ ^71^ ^]^ Afterwards, they achieved polymer crystal microwires with controlled assembly from polymer solutions.^[^ ^72^ ^]^ It has also been shown that the backbone conformation of conjugated polymers plays a significant role in interchain aggregation, where twisted chains usually result in weak interchain interactions and reduced aggregation.^[^ ^73^, ^74^ ^]^ Jie and coworkers reported the precise patterning of organic p−n heterojunction arrays of conjugated small molecules for ambipolar field‐effect transistors.^[^ ^42^ ^]^ They obtained large‐scale, aligned, and precise patterning of anthradithiophene and perylene diimide based molecules (dif‐TES‐ADT and BPE‐PTCDI) by surface‐energy‐controlled stepwise crystallization using prepatterned photoresist stripes (Figure 5d). During the crystallization, the molecules would transport to the meniscus front while the photoresist stripes provided high‐surface‐energy sites. Dif‐TES‐ADT molecules initially crystallized at the edges forming the crystalline microbelts. Then, dif‐TES‐ADT crystals served as new high‐surface‐energy sites to induce the assembly and crystallization of BPE‐PTCDI. These surface‐energy‐controlled stepwise crystallization supports the formation of orderly stacked p‐n microbelts (**Figure** **6**e–g). Furthermore, Jie and coworkers demonstrated field‐effect transistors with high mobilities and inverters with large voltage gains by utilizing the p−n microbelts (Figure 5h). This surface microstructure‐assisted patterning will be useful to fabricate large‐scale and flexible integrated organic circuits.^[^ ^75^ ^]^

### 2.2 Nanoscale to Microscale Order: Lithography‐Based Approaches toward Forming 2D Patterns

In recent years, it is becoming increasingly common to introduce 2D ordered patterns of organic electronic components via lithographic techniques. Optical lithography can be used for generation of larger‐scale patterns, while E‐beam lithography, nanoimprint lithography, and scanning probe lithography among others can be used to generate nanoscale patterns (Figure 1c).^[^ ^28^ ^]^ Patterning of electroactive organic materials is crucial in the processing of integrated circuits and is the basis for the fabrication of high‐performance organic electronic components that are becoming more relevant nowadays since more flexible devices are being commercialized. However, organic molecular materials are difficult to pattern using approaches such as photolithography and chemical etching that are conventionally designed for silicon‐based hard inorganic materials. Organic materials are usually soluble or swellable in organic solvents due to weak non‐covalent intermolecular interactions. Therefore, patterning organic semiconductors usually requires an appropriate orthogonal solvent which can be used in photolithography and chemical etching and does not damage organic semiconductor layers. Traditionally, patterning of organic semiconductors is achieved by using solution‐processing, such as inkjet printing or nanoimprinting.^[^ ^76^, ^77^, ^78^ ^]^ Direct and precise patterning of organic molecular materials still remain challenging.

To solve some of the issues for the direct patterning of conjugated polymers, Kim and Lee et al. developed a 3D crosslinker (4Bx) with four photocrosslinkable arms based on azide groups (Figure 6). The developed crosslinker, 4Bx, can be well mixed with solution‐processable electroactive organic materials, such as conjugated polymers and insulating polymers. The four azide groups in 4Bx can provide highly reactive singlet nitrene after UV irradiation and then crosslink the neighboring alkyl chains by insertion reactions into the C—H bonds (Figure 6b). Based on this crosslinking mechanism, the mixed polymer systems with crosslinker molecules could generate well‐crosslinked networks after UV irradiation since 4Bx has four crosslinking sites in one molecule (Figure 6a). Importantly, the crosslinked layer is highly resistant to chemical solvents, supporting the patterning process with a high resolution approaching 10 µm. They demonstrated the designed patterning of conjugated polymers such as P(DPP2DT‐TVT). Furthermore, benefitting from the orderly patterned conjugated polymers as the active layer and PMMA as the dielectric layer, they successfully obtained all‐photopatterned polymer field‐effect transistors and logic circuits (Figure 6d). This work demonstrated an effective method to fabricate integrated electronic devices of conjugated polymers based on a simple 3D crosslinker. On the other hand, Zheng and Yu et al. employed the azide crosslinking chemistry to form the covalently‐embedded in situ rubber matrix (iRUM), which can mix well with conjugated polymers and form crosslinked networks.^[^ ^79^ ^]^ The iRUM precursors can covalently crosslink the alkyl chains in conjugated polymers and insulated polymers, providing high crosslinking density and hence superior elasticity and solvent resistance. Therefore, they demonstrated the azide crosslinking chemistry could be used in precise full patterning for stretchable transistors. The stretchable transistors based on iRUM‐semiconductor showed robust mobilities over 1 cm^2^ V^−1^ s^−1^ after 1000 stretching‐releasing cycles at 50% strain.

Another novel and efficient crosslinker based on diazirine groups by employing carbene insertion was developed by Zheng, Liu, Zhong, et al. (Figure 6e).^[^ ^38^ ^]^ The diazirine groups could form carbene and could insert into C—H bonds within the neighboring alkyl chains,^[^ ^81^ ^]^ further forming the covalent crosslinked networks. This crosslinker demonstrated excellent compatibility for different conjugated polymers and insulated polymers. They achieved 2‐µm resolution line patterns for PEDOT:PSS and 4‐µm resolution line patterns for DPPTT through the diazirine‐based crosslinker and direct optical lithography (Figure 6f,g). Therefore, they were able to fabricate transistor array with a high device density of 42,000 transistors per cm^2^ and a small transistor channel length of 2 µm. At the same time, the diazirine‐based crosslinker for patterning conjugated polymers has also been reported by Zhang and coworkers.^[^ ^82^ ^]^ They achieved 5‐µm resolution patterns of several different conjugated polymers using a diazirine‐based four‐armed crosslinker. Based on this direct patterning method, they demonstrated an integrated inverter using patterned n‐ and p‐type conjugated polymers and achieved a high gain value of 112. Overall, these crosslinker‐based lithography methods provide efficient and precise patterning approaches with designed order and high resolution down to several micrometers of conducting polymers. These strategies are also compatible with the biomaterials in principle and will be useful to fabricate integrated circuits of electroactive organic molecular materials.

**Figure 7:**
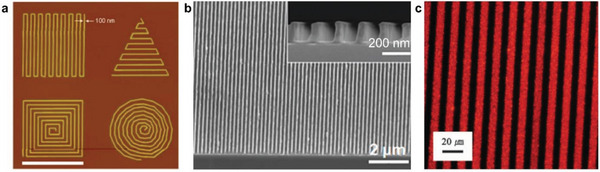
Multi‐scale patterning of polymers. a) AFM image of pSS*‐co‐*PEGMA patterns by E‐beam lithography. Reproduced with permission.^[^ ^37^ ^]^ Copyright 2008, ACS. b) SEM image of P3HT patterns by nanoimprint lithography. Reproduced with permission.^[^ ^83^ ^]^ Copyright 2009, ACS. c) Fluorescence microscope image of PDA patterns by micromolding in capillaries. Reproduced with permission.^[^ ^84^ ^]^ Copyright 2008, WILEY‐VCH Verlag GmbH & Co.

Photolithography‐based patterning methods usually require prepatterned masks to transfer the patterns onto the photoresist layer, adding another layer of intricacy into the process. Direct patterning methods without prepatterned masks can be easily approached although the patterning process might take longer time due to point‐by‐point exposure, like laser writing and etching and E‐beam lithography. DeForest and coworkers developed a photocleavable linker based on a photoinduced *β*‐elimination reaction for patterned protein release from biomaterials.^[^ ^49^ ^]^ E‐beam lithography is a technique to pattern nanoscale features without requiring a photomask.^[^ ^32^ ^]^ In this technique, an E‐beam is focused on a substrate coated with a polymeric thin film, causing chemical changes that will either crosslink or degrade the polymeric thin film.^[^ ^32^ ^]^ For example, in 2008, Christman et al. reported the direct E‐beam lithography of a blocking polymer, leading to conjugation of proteins to polymer nanopatterns.^[^ ^37^ ^]^ They patterned the polymer surface of poly(sodium 4‐styrenesulfonate‐*co*‐poly(ethylene glycol) methacrylate) (pSS‐*co*‐pPEGMA) with nanoscale resolutions down to 100 nm (**Figure** **7**a). Therefore, proteins of basic fibroblast growth factor and vascular endothelial growth factor could bind to the patterned polymer surface via the heparin‐binding domains. While E‐beam lithography has many advantages such as ability to generate patterns with small inter‐feature spacing, this lithography technique is more expensive and slower compared to photolithography.^[^ ^32^ ^]^ In order to pattern biomolecules, E‐beam lithography can also locally alter hydrophobicity and functionality of polymer resists, allowing for biomolecules to be patterned via hydrophobic interactions or coupling chemistries, or E‐beam lithography can be utilized to degrade regions of a protein monolayer for further adsorption with a second protein.

Nanoimprint lithography is a more cost‐efficient and high‐throughput fabrication approach compared to E‐beam lithography, while also maintaining a high patterning resolution.^[^ ^28^, ^78^, ^83^, ^85^ ^]^ Importantly for organic photovoltaics, nanoimprint lithography‐based polymer chain alignment has been shown to improve charge carrier transport in conjugated polymers as well as enhance light adsorption and charge collection.^[^ ^78^ ^]^ In the fabrication of the organic photovoltaics, a hard mold with nano‐scale structures treated with an anti‐adhesion layer and the polymer of interest are brought into contact with each other.^[^ ^78^, ^85^ ^]^ With the addition of pressure and heat—typically ≈50 °C higher than the polymer of interest's glass transition temperature—the polymer will convert to a viscous state and flow into the mold cavities.^[^ ^78^ ^]^ This technique has been utilized by Ding et al. to create large area poly(3‐hexylthiphene) (P3HT) nanopillar arrays and induce a *π*−*π* stacking alignment normal to the substrate which is favorable for photovoltaic cell applications.^[^ ^85^ ^]^ Similarly, Aryal, et al. employed the geometry‐dependent confinement during nanoimprinting to order and align P3HT nanostructures (Figure 7b) and improve charge transport and optical properties of solar cells and field effect transistors.^[^ ^83^ ^]^ A unique family of conjugated polymer, polydiacetylenes (PDAs) have also been patterned via a variety of methods, including photolithography and a technique based on micromolding in capillaries (MIMIC) (Figure 7c).^[^ ^84^, ^86^ ^]^ Indeed, PDAs have found an important use in biosensing applications and photonic materials due to a visible color transition between blue‐ and red‐colored PDAs in response to environment stimulation, and as such there has been considerable demand for developing an efficient methodology for immobilizing PDA vesicles into micropatterns.^[^ ^84^, ^86^ ^]^ The MIMIC technique involves leveraging capillary action to move the PDA solution through a poly(dimethylsiloxane) (PDMS) mold on a substrate, followed by the evaporation of the PDA solvent and the eventual UV polymerization of the PDA. In another exemplar work, Choi et al. devised a strategy to pattern PDA using pre‐patterned fluorocarbon hydrophobic thin films on glass substrates to selectively immobilize PDA vesicles, which showed significant fluorescence response for sensing different cyclodextrins.^[^ ^86^ ^]^

### 2.3 Etch‐Based Printing

Multi‐scale patterning of electroactive and photovoltaic materials has also been established in ordered structures via a top‐down, etching‐based printing techniques, such as plasma etching, chemical etching, inkjet etching, and laser etching.^[^ ^30^, ^87^, ^88^ ^]^ An inkjet printer can be utilized for a subtractive etching process, known as inkjet etching.^[^ ^87^ ^]^ This process involves dropping a single droplet of solvent on a pre‐deposited polymer film, resulting in a ring‐like deposit through what is known as a coffee‐ring effect. The outward convective flow of the polymer solution during solvent evaporation results in the redeposition of the polymer outward and away from the initial droplet impact site, leading to a crater‐like deposit. In some cases, successive dropping of solvent can be utilized in situations where single droplets are unable to etch the polymer. These inkjet‐etched polymers can be utilized for organic electronic devices, including as optical waveguides as well as platforms for selective deposition of organic materials. Laser etching remains another popular patterning technology and has in recent years leveraged the conductivity, flexibility, and biocompatibility of graphene as a strategy to produce supercapacitors, optoelectronics, and electro‐actuators.^[^ ^88^ ^]^ Direct laser writing techniques allow for the desired patterns to be made without the use of a mask or chemicals; however, this process requires point‐by‐point exposure and thus is more time consuming than UV lithography approaches. In the case of graphene, its properties vary with the number of layers, and as such, laser etching can be used to controllably thin the top layer of graphene into specified patterns. Inductively coupled plasma reactive ion etching (ICP‐RIE) is another technique used in the fabrication of semiconductor technologies.^[^ ^89^ ^]^ This selective, dry‐etching technique can introduce non‐planar microstructures, including tilted sidewalls with a specified angle, and is more commonly used with harder substrates such as silicon carbide. ICP‐RIE involves the interactions of ions with the etched material as the result of chemically active radicals, ionized atoms, excited atoms, undissociated atoms, and free electrons within plasma. Indeed, ICP‐RIE combines physical and chemical reactions, with chemical reaction involving the surface of the etched material being converted to volatile products from the free radicals of the plasma, while the physical reaction is a high energy ion bombardment. This balance between the physical and chemical mechanisms can be tuned to optimize the ICP‐RIE process for specific masks and substrates.

### 2.4 Tailoring the Micro‐ to Macroscale Architecture via Additive Manufacturing

Additive manufacturing techniques for ordering the micron to macroscale architecture of electroactive polymers and biomolecules with the bottom‐up method can be divided into “drop‐by‐drop,” “line‐by‐line,” and “layer‐by‐layer” based on the patterning process. For the “drop‐by‐drop” process, inkjet printing is one of the earliest technologies developed for accurate patterning.^[^ ^76^ ^]^ The materials must be either dissolved or dispersed in solution as “ink” and then ejected to the substrate through the nozzle.^[^ ^76^, ^90^, ^91^ ^]^ The programming of nozzle position for material ink‐drop position enables different complex architectures of electroactive polymers and biomolecules homogeneously.^[^ ^92^, ^93^ ^]^ Also, the resolution of patterning can be improved by optimization of several parameters, such as the nozzle size, the viscosity and evaporation rate of “ink,” and the movement speed of nozzle.^[^ ^94^, ^95^ ^]^ Thus, inkjet printing has been applied to the development of organic electronics for designed 1D or 2D structures with versatile electroactive polymers as ink to facilitate charge transport.^[^ ^96^, ^97^, ^98^ ^]^ However, there are still several challenges for complex patterning with inkjet printing. Although higher resolution may be achieved by thinner nozzles, it is hard to reach patterning resolution higher than the micrometer level. In addition, the thinner nozzles are more likely to induce clogging of polymer solution with high viscosity and biomolecule solution/dispersion with a high concentration. This will reduce the reliability and repeatability of the process.^[^ ^99^ ^]^ Especially, the drop‐by‐drop printing method may hinder the continuity between patterns, even lines, due to the interaction between inks and substrate, such as contact angles and surface tension. Furthermore, inkjet printing is limited to only 2D patterning due to the confinement of nozzle movement in the x‐y plane, hindering its application in building biomimetic 3D extracellular matrix for tissue engineering.

**Figure 8:**
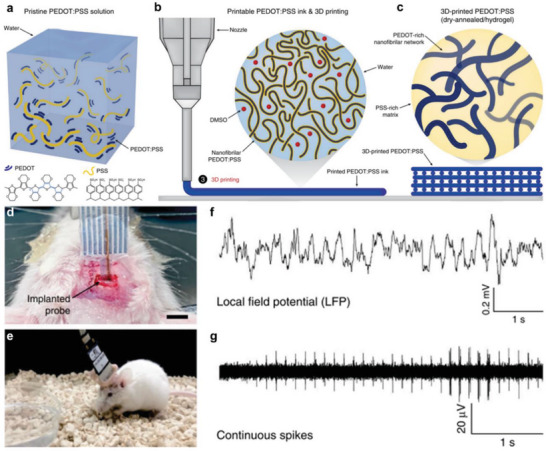
3D‐printed soft neural probe using a conducting polymer of PEDOT:PSS. a–c) Design strategy of 3D‐printable conducting polymer ink of PEDOT:PSS. d) Implanted 3D‐printed soft neural probe. e) A freely moving mouse with an implanted 3D‐printed soft neural probe. Electrophysiological recordings in the mouse dHPC by the 3D‐printed soft neural probe under freely moving conditions: f) Local field potential (LFP) traces (0.5 to 250 Hz). g) Continuous extracellular action potential (AP) traces (300 to 40 kHz). Reproduced under the Creative Commons Attribution 4.0 license.^[^ ^100^ ^]^ Copyright 2020, The Authors. Published by Springer Nature.

To construct more continuous patterns with 3D architectures, extrusion‐based 3D printing with a line‐by‐line process has raised increasing attention due to its flexibility and efficiency in macroscale patterning.^[^ ^101^, ^102^, ^103^ ^]^ Other than drop‐by‐drop deposition, the ink flow of electroactive polymer and biomolecules is continuously extruded to the substrate in a line‐by‐line manner.^[^ ^101^, ^104^ ^]^ The critical point for improving the pattern integrity is maintaining the ink deposition at the designed location, not flowing to the surroundings to overlap or interrupt each other. Optimizing the viscosity of inks is one of the methods to stabilize the structures for homogeneous and complex patterning.^[^ ^105^, ^106^ ^]^ To our knowledge, Yuk and Lu et al. were the first to succeed in the 3D printing of electroactive conjugated polymer, poly(3,4‐ethylenediox‐ythiophene): polystyrene sulfonate (PEDOT:PSS), by tuning its concentration and gel formation (**Figure** **8**).^[^ ^100^ ^]^ The optimized concentration of PEDOT: PSS not only facilitated the formation of printable soft hydrogel due to the suitable and ordered entanglements within DMSO/water to avoid the spreading of PEDOT: PSS on the substrate, but also enhanced the alignment of PEDOT: PSS nanofibril from the shear force to improve the conductivity (Figure 8a–c). The quick drying process of DMSO and water enables the 3D patterning of multi‐layered structures and fosters PEDOT‐rich crystalline domains formation for higher conductivity (Figure 8c). In another work, a parallel approach was demonstrated by mold casting hydrogels composed of water‐soluble EDOT‐diethylene glycol that are electronically conductive upon in situ polymerization and can maintain the mechanical properties of the gel.^[^ ^107^ ^]^ In the future, a better understanding of polymer chain entanglements and formation of nanofibril networks in PEDOT‐based or PEDOT:PSS solutions will advance the development of these 3D printable polymer systems. Moreover, the flexibility and reproducibility of 3D printing of conductive PEDOT: PSS allows the fabrication of bioelectronic signal recording probe with PDMS (Figure 8d,e). The success of implantation in mice and continuous neural activities recording provides the potential of using electroactive and biocompatible polymers for neuroscience and behavior studies (Figure 8f,g).

**Figure 9:**
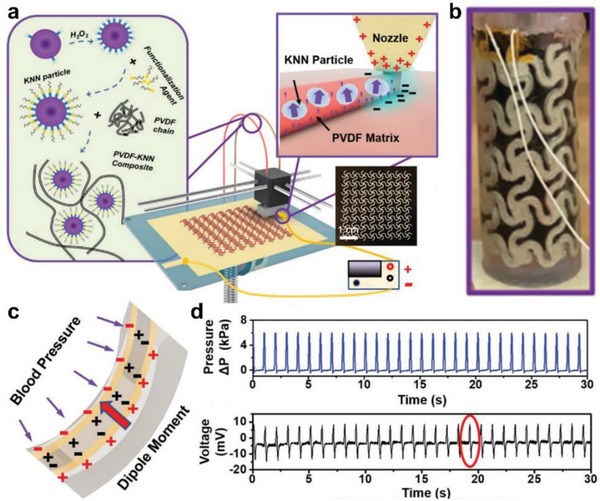
3D‐printed multifunctional artificial arteries using PVDF‐KNIN composite. a) Schematic illustration of the electric field‐assisted 3D printing system using PVDF‐KNN ink. b) Optical image of a 3D‐printed artificial artery. c) Schematic illustration of the piezoelectric effect in artificial artery in response to blood pressure. d) Real‐time change of pressure and voltage output using the 3D‐printed artificial artery under PBS‐simulated blood flow. Reproduced with permission.^[^ ^39^ ^]^ Copyright 2020, WILEY‐VCH Verlag GmbH & Co.

On the other hand, higher viscosity of inks can induce higher shear force during the extrusion process, which may either interrupt the polymers network structure, affecting its electrical properties such as conductivity, or reduce cells viability when cells are mixed with biomolecules in media as bioink.^[^ ^108^, ^109^ ^]^ Particularly, cells that are sensitive to mechanical force will alter their differentiation and proliferation activities due to the changed physical properties of electroactive polymer or biomolecule solutions. To solve this problem, supramolecular chemistry has been applied to develop ink with reversible mechanical properties as the result of non‐covalent interactions, such as host‐guest interactions and hydrogen bonding.^[^ ^110^ ^]^ External cues, such as light, temperature, electrical fields, and magnetic fields, have been utilized for rapid polymerization to stabilize the inks on the substrate. Light‐induced polymerization can achieve spatiotemporal control for accurate control of patterns with complex structures. For example, co‐shell, heterogeneous, and hollow structures with different materials can be patterned by controlling the exposure part in nozzle to the light.^[^ ^111^ ^]^ Moreover, cell suspension can be homogeneously mixed with monomer solutions and patterned simultaneously to build more biomimetic in vitro tissue models. The electric field has also been applied to improve 3D printing for functional patterning, especially with piezoelectric materials. Li et al. coupled the concentrated adjustable electric field to the extrusion‐based 3D printer between the nozzle and substrate platform (**Figure** **9**).^[^ ^39^ ^]^ Consequently, the homogeneous filament of 35% piezoceramic sodium potassium niobate (KNN) and 65% PVDF polymers can be patterned using 3D printing (Figure 9a,b). The dipoles in the piezoelectric polymer of PVDF can be aligned simultaneously during the 3D printing process, supporting its sensitivity to external pressure (Figure 9a–c). In addition, the patterned architecture increased the functional surface area to transform the blood pressure to the electro signals. Furthermore, the demonstrated linearity and sensitivity of the PVDF/KNN composite enable the artificial arteries with self‐powered sensing capability, biocompatibility, and reliability for monitoring real‐time blood pressure. The designed artificial arteries successfully showed a significant voltage response to the blood flow pressure (Figure 9d). This work demonstrates an excellent example of using 3D printed materials with specific patterns to achieve multiple functionalities. Furthermore, based on this work, we envision that combining extrusion‐based 3D printing with electro‐spinning techniques will improve the nanofiber structures of patterned hydrogels to enhance the functionalities such as conductivity and cell adhesion.^[^ ^112^, ^113^ ^]^

To further increase the efficiency and continuity, layer‐by‐layer patterning techniques have been developed. Microcontact printing can directly pattern desired electroactive polymers and biomolecules to the substrate. However, the molds with different geometry must be preprepared, reducing the ease of use and possibilities for large‐scale fabrication. A digital micro‐mirror device‐based system or digital light processing (DLP)‐based printing techniques were proposed by Chen and coworkers to achieve 3D patterning with a layer‐by‐layer process.^[^ ^114^, ^115^ ^]^ For DLP‐based 3D printing, UV‐light is first reflected by a digital micromirror device (DMD) chip and then projects an optical pattern to the photo‐induced polymer solutions on the substrate. Electroactive polymers and biomolecules can be patterned to any complex geometry by applying the optical patterns designed by computer‐aided design. In addition, the layer‐by‐layer patterning process for 3D architectures can be achieved by projecting the whole optical pattern plane to the substrate and switching the substrate height. Compared to the extrusion‐based drop‐by‐drop or line‐by‐line process, this DLP‐based layer‐by‐layer patterning increases the patterning speed significantly compared to the drop‐by‐drop or line‐by‐line process. The complex 3D structures of honeycomb built by microtubule structure and mushroom‐like array with 1‐µm resolution were achieved with piezoelectric polymers composite materials with barium titanate nanoparticles covalent with polyethylene glycol diacrylate (PEGDA) matrix, showing the potential to generate different functional surface for electronic applications.^[^ ^116^ ^]^ The functionalized patterning can be made with heterogeneous materials to control the patterned models with external stimuli, such as electrical fields, chemical fuels, and magnetic force.^[^ ^117^ ^]^ Since the mechanical properties of ink will retain throughout the entire process, the ink candidates of materials, especially biomolecules, are not limited by their rheology properties. Hence, the patterning for cells together with polymers and biomolecules can be completed without sacrificing cell viability.^[^ ^118^ ^]^ Meanwhile, the physical properties such as stiffness of the patterned microenvironment can be easily tuned by changing polymerization time by light exposure time, providing a high throughput method to form ordered structures.

## 3 Controlling Conduction across Length Scales in Electrogenic Biological Systems

In the previous sections, we have established various tools and approaches for engineering the structural organization of synthetic and bio‐inspired electroactive organic materials. We presented previous works that direct multi‐scale ordering to improve charge transport properties or impart 2D/3D patterns to dictate high resolution conductive pathways for biologically relevant organic electronic devices. The subsequent sections focus on the requirement of structural organization on electroactive or electrogenic living and biotic‐abiotic hybrid living systems, and how these can be achieved via some of the fabrication approaches discussed above. The discussions below will highlight the recent progress towards achieving long‐range order in biological and biohybrid systems to control the functionalities of bioelectronic devices and platforms.

### 3.1 In Vitro Cellular Patterning

Micro and nanoscale cellular order in vitro is critical to cell function and behavior, as well as for controlling cell shape and spreading for cell‐based biosensors and other tissue engineering‐based devices.^[^ ^119^, ^120^, ^121^, ^122^ ^]^ Micropatterning techniques are pivotal in stem cell research to control cellular order, where commitment to specific lineages is dependent to cell shape and microenvironments. To achieve control over cellular morphology and tissue order, various patterning techniques have been proven useful, including plasma etching, photolithography, and micro‐contact printing. Microcontact printing, an extension of photolithography techniques, is one of the most widely used approaches to geometrically confine cells in micropatterns and also control electrical signaling via cell‐cell communication. To improve the efficiency of the patterning techniques and prevent non‐specific cell adhesion on a substrate, non‐fouling surface modifications are used; poly(ethylene glycol) (PEG) is one of the most used non‐fouling surfaces due to its protein resistance and ability to reduce protein adsorption. These micropatterned platforms have found use with a range of cell types, from hepatocytes to adipose derived stem cells. Hepatocytes have been a popular target for cellular patterning techniques to mimic the complex microenvironment of the in vivo liver. For example, Otsuka et al. developed an in vitro system to improve hepatocyte viability outcomes and create a functional, miniaturized liver by utilizing dry‐etching techniques to design a 2D microarray system of ten thousand hepatocyte spheroids underlaid with endothelial cells.^[^ ^123^ ^]^ Yu et al. utilized microgrooves to increase substrate hydrophobicity, and greatly improved adipose derived stem cell adhesion and proliferation, introducing and aligning directional growth of the cells along the groove.^[^ ^124^ ^]^

**Figure 10:**
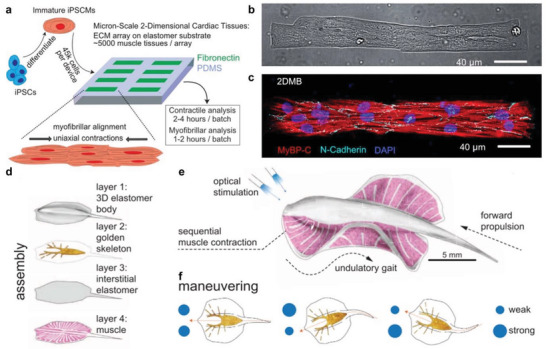
a–c) Microcontact patterning of cardiac muscle bundles. a) Schematic diagram of the approach using microcontact patterned fibronectin to pattern cardiac muscle bundles. b) Brightfield microscopy image of micropatterns showing hPSC‐CMs avidly adhere and conform to PDMS micropatterns but not to surrounding PDMS to generate 2DMBs. c) Myofibrils (marked by MyBP‐C) develop along the 2DMB long‐axis traversing continuously across cell junctions (marked by N‐cadherin). Reproduced under the Creative Commons Attribution 4.0 license.^[^ ^126^ ^]^ Copyright 2021, The Authors. Published by Springer Nature. d–f) Tissue‐engineered ray. d) Order of the four‐layer body architecture; e) concept diagram; f) phototactic controlled movement. The patterned muscle tissue is optically stimulated, creating an undulatory swimming motion. Reproduced with permission.^[^ ^128^ ^]^ Copyright 2016, AAAS.

In microcontact printing, a silicon wafer with the master pattern is developed using photolithography; and PDMS is used to create a replica mold “stamp” that can be coated in a polar ink.^[^ ^125^ ^]^ After allowing the ink to incubate and coat the surface of the patterned PDMS, the coated stamp can then be used to transfer the pattern onto a desired surface.^[^ ^125^ ^]^ For example, Tsan et al. utilized micro‐contact printing to develop in vitro cardiac tissues with fibronectin as the ink to promote cellular adhesion (**Figure** **10**a–c).^[^ ^126^ ^]^ In this work, individual µm‐scale micropatterns were fabricated using micro‐contact printing with a buffering distance to mechanically decouple adjacent 2D cardiac muscle bundles (Figure 10a). They found that the cardiac muscle could grow ordered bundles on the 2D elastomer substrates (Figure 10b,c). Furthermore, cardiac muscle bundles formed significant myofibrillar alignment and exhibit uniaxial contractions. Briefly, this work shows that patterned surfaces can be used to develop desired tissues. For cardiac in vitro models, the organization of cells dictates the pathway of electrophysiological signals (i.e., Ca^2+^ propagation), which impacts higher order function such as the excitation‐contraction coupling in cardiac tissues that controls their mechanical motion.^[^ ^127^ ^]^ For example, by imparting a serpentine bioadhesive protein micropattern on an elastomeric substrate, Parker and coworkers reported a biohybrid system with a sting ray macro‐morphology that is built with patterned rat cardiomyocytes and capable of a photo‐guided propulsion in aqueous solutions (Figure 10d–f).^[^ ^128^ ^]^ They successfully fabricated the artificial body architecture with a four‐layer order of 3D elastomer body, golden skeleton, interstitial elastomer, and optogenetically modified rat cardiomyocytes that are micropatterned (Figure 10d). Optical stimulation of this muscular pump model allowed for a sequential muscle activation, leading to photo‐guided undulatory swimming motions recapitulating that of naturally existing sting rays (Figure 10e). Calcium imaging revealed that photostimulation of the circuit triggered the movement and redistribution of ions between cardiomyocytes, thereby controlling muscular contraction down to the cellular level and bringing macroscopic photo‐guided swimming movements.

Micropatterning techniques have also been leveraged to advance various aspects of neuronal cell development and better mimic the in vivo neuronal microenvironment.^[^ ^3^, ^129^, ^130^, ^131^, ^132^ ^]^ Orientation of neuronal cell growth is especially important as the directional growth is imperative to form neuronal growth between neighboring neurons. Schmidt and coworkers reported the µm‐scale patterning of a typical conducting polymer, polypyrrole (PPy), to create electrical and topographical cues for the cell stimulations.^[^ ^133^ ^]^ They fabricated well‐patterned 1‐ and 2‐µm microchannels of PPy using E‐beam lithography and electropolymerization. Embryonic hippocampal neurons cultured on patterned PPy could polarize more rapidly than was the case on unmodified PPy.^[^ ^133^ ^]^ Fan et al. fabricated 3D anisotropic micropillar scaffolds using direct laser writing in order to guide the orientation of axon and dendrite growth.^[^ ^134^ ^]^ The micropillars within the scaffold were of a range of spacings and heights, reducing neurite branching, encouraging axons and dendrites to form neuronal circuits, and promoting synchronous action potential firing patterns. In a different approach, micropatterning has been introduced to improve the ability of bioelectronic neural interfaces to facilitate communication between patient's nerve fibers and therapeutic computer platforms.^[^ ^135^ ^]^ In particular, fibroblast have been established to trigger foreign body responses in these bioelectronic neuronal interfacing technologies and result in fibrotic encapsulation and worsen neural tissue integration. Hence, Mobini, et al. identified a specific polyimide micropattern that can promote Schwann cell activity and inhibit fibroblast growth to address this current limitation in bioelectronic neural interfacing technology.^[^ ^135^ ^]^

### 3.2 Scaffold‐Templated Tissue Engineering

Scaffolding of cells and tissues using engineered biomaterials is a widely used technique in 2D and 3D tissue engineering. To control the global order of constituent electroactive cells that populate a bioscaffold, the abiotic components should be biocompatible and have favorable physical properties that will allow cells to comply with physicochemical cues that will enable their microscale organization.^[^ ^136^, ^137^, ^138^ ^]^ Biocompatible or bioinert electroactive polymers, such as PPy and polyaniline can offer such properties to stimulate the facilitation of the biochemical cues for tissue growth.^[^ ^3^, ^113^ ^]^ Moreover, the electroactive functionalities enable the direct stimulation to tissues in a spatiotemporal manner by applying electric fields. Thus, tissue behaviors can be controlled or studied for various purposes, such as real‐time sensing and therapy, with minimum invasion. Furthermore, the ordered 3D tissue structures by utilizing the patterning techniques described above can influence extracellular matrices or scaffolds to become more biomimetic for biomedical applications such as implantation.

Cellular behavior studies visualizing cellular response to altered microenvironments can benefit from 3D tissue engineering with complex patterning. With 3D printing techniques, heterogeneous extracellular matrix with different physical properties, such as stiffness, can be easily patterned. Ma and Yu et al. pattered decellularized extracellular matrix with different stiffnesses in order to visually study live cell migration and growth differences.^[^ ^139^ ^]^ Later, iPSC‐derived hepatic cells, endothelial‐ and mesenchymal cells encapsulated in the ordered hepatic lobule structure were patterned together, mimicking physiological microenvironment and microarchitecture, to study the hiPSC hepatic differentiation.^[^ ^140^ ^]^ Gjorevski, Nikolaev, and Brown et al. realized accurate control of mouse intestinal organoids orders in terms of the location, initial size, and shape by patterning, providing a platform to study the number and location of crypt‐like domains during organoids development.^[^ ^141^ ^]^ Although the electro‐related properties have not been studied, electroactive organic molecular materials can be easily included in the studying of cell behaviors such as migration and differential with electric fields.^[^ ^17^ ^]^

Since vascularization is one of the most essential processes for tissue regeneration, such as functional cardiac tissue and bone formation, patterning ordered vascular tissues with necessary growth factors and complex structures to improve nutrient transportation and bio‐signal conduction has been widely studied.^[^ ^142^ ^]^ Zhu and Qu et al. patterned revascularized tissues encapsulated by 3D microarchitectures with gradient channel widths to simultaneously mimic the native vascular cell microenvironment and observe the functional vasculature formation.^[^ ^143^ ^]^ Piezoelectric materials, such as KNN and PVDF, have already been 3D printed as artificial arteries with the sinusoidal lattice to mimic the mechanical modulus to the level of blood vessels. With this ordered structure through an electric field‐assisted 3D printing, the performance of piezoelectric materials in converting blood pressure to electrical signals has been demonstrated.^[^ ^39^ ^]^ Thus, including piezoelectric materials provides the potential for real‐time study of blood pressure in vascular tissue models. Since cardiac disease is one of the leading factors for human death, patterning 3D cardiac tissues is an essential tool for disease studies, drug development, and facilitating tissue maturation.^[^ ^144^ ^]^ Liu et al. patterned neonatal mouse ventricular cardiomyocytes along with different complex structures to study how different geometries can affect the contractile force and generate cardiac disease models for tissues such as the myocardium.^[^ ^145^ ^]^ The relation between patterned scaffold displacement and force was demonstrated in this work by taking advantage of using electroactive polymers that can not only transfer force to electrical signals with piezoelectric polymers but also influence cardiac differentiation by employing electrical stimulations with varied frequency. For example, Shin et al. proposed conductive granular hydrogels which can be patterned to a lattice,^[^ ^146^ ^]^ where the patterned lattice was implanted on porcine myocardium and bridged tissues. Dlay et al. patterned cardiomyocyte and fibroblast cells with hydrogel for a 3D cardiac tissue model and observed the reduced contractility and electrical activity.^[^ ^108^ ^]^ The electrical signal transferred between different cells or tissues can be studied by incorporating the mentioned conductive granular hydrogels. Interestingly, human‐derived cardiomyocytes have previously been patterned in biohybrid fish construct to achieve self‐sustained swimming via the spontaneous contractile nature of cardiomyocytes enhanced by an electrically autonomous pacing node.^[^ ^147^ ^]^

Other than in vitro 3D tissue scaffolds, patterning electroactive polymer and biomolecules has also inspired researchers to investigate potential in vivo biological applications such as sensing and therapy.^[^ ^21^, ^148^ ^]^ Koffler and Zhu et al. succeeded in 3D printing hydrogel scaffolds with neural progenitor cells.^[^ ^149^ ^]^ The neural progenitor cells were precisely patterned to various sizes and dimensions for practical implantation. The neural progenitor cells synapsed with injured host axons and thus improved central nervous system regeneration. In addition, the precise shape and dimensions of patterned hydrogel scaffolds with neural progenitor cells achieved by 3D printing potentiate the precision medicine for various biomedical implantation requirements. Although this work has already achieved unprecedented progress in spinal cord injury repair by patterning, various functional materials other than glycol–gelatin methacrylate can be applied to further facilitate recovery from spinal cord injuries, such as IKVAV peptide amphiphile and FGF2 peptide amphiphile.^[^ ^150^ ^]^ Previous reports have shown that electroactive biocompatible polymers are possible to be applied for the optoelectronic excitation and inhibition of neural activities.^[^ ^151^, ^152^ ^]^ To achieve the fine control of patterned architectures of scaffolded electroactive cells on life‐size in vitro models, Chang, Liu, and Zimmerman et al. applied the focused rotary jet spinning to form the fine spatial features with controlled helical and circumferential alignments. The formed left ventricle models seeded with cardiomyocytes demonstrated the robust biomechanical features resembling theoretical findings.^[^ ^112^ ^]^ Furthermore, 3D printing has been developed even for tissue constructs in vivo by noninvasive methods. For example, Chen and Zhang et al. injected biocompatible hydrogel monomers into mice and induced spatial polymerization ex vivo using near‐infrared light to directly pattern monomers to ear‐like tissue, indicating its potential for organ regeneration therapy.^[^ ^153^ ^]^ Although this work focuses on the ordered structures through 3D printing, we believe that electroactive materials can be applied in these printed structures to stimulate signaling and enhance specific functions.

### 3.3 Biotic‐Abiotic Interfacing for Device Applications

**Figure 11:**
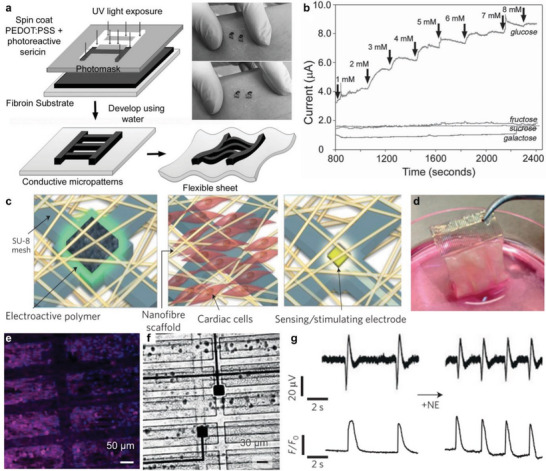
Biosensors with patterned electroactive organic materials. Microelectronic cardiac patch. a) Schematic diagram of fabricating conductive micropatterns on a flexible substrate. b) Current response of PEDOT:PSS against glucose addition while fructose, sucrose, and galactose served as control experiments. Reproduced with permission.^[^ ^23^ ^]^ Copyright 2016, WILEY‐VCH Verlag GmbH & Co. c) Concept diagram for a microelectronic cardiac patch. d) Image of the folded microelectronic cardiac patch after 7 days of cultivation with cardiac cells. e) Confocal microscope image of the assembled cardiac tissue within the hybrid electronic biomaterial. Magenta: sarcomeric actinin; blue: nuclei. f) Brightfield microscopy image of a folded device depicting distant electrodes. g) Recordings of cellular electrical activity (top) in parallel with fluorescence intensity quantification of calcium transients (bottom) before and after the addition of norepinephrine (NE). Reproduced with permission.^[^ ^24^ ^]^ Copyright 2016, Springer Nature.

Biotic‐abiotic interfacing for device applications require consideration of the biocompatibility of abiotic systems, including toxicity, mechanical properties, ion concentration, etc.^[^ ^17^, ^154^, ^155^ ^]^ Conductive polymers, such as PPy, polythiophenes, and PEDOT:PSS, demonstrate excellent biocompatible, suggesting their potential application in tissue engineering, biosensors, or development of electronic skin.^[^ ^156^, ^157^, ^158^, ^159^, ^160^, ^161^, ^162^, ^163^, ^164^ ^]^ Sensing and converting electrical signals is one of the important biotic‐abiotic interfacing applications of electroactive materials in the biomedical space.^[^ ^15^, ^165^, ^166^, ^167^ ^]^ In this section, we describe the progress on ordered and patterned electroactive biotic‐abiotic interfaces used in biomedical devices, particularly toward ensuring that the biocompatibility of the abiotic components is maintained throughout the fabrication process. The work of Yadavalli et al. represents an example of a simple patterning method to fabricate flexible and biodegradable devices via an aqueous photolithographic process of silk proteins and a conducting polymer of PEDOT:PSS (**Figure** **11**a,b).^[^ ^23^ ^]^ Photoreactive sericin was blended with PEDOT:PSS solutions and finally resulted in a photopatternable and conductive ink. This conductive ink could form homogeneous films on substrates by solution spin‐coating process. Crosslinking with 365‐nm UV exposure, following development in water could lead to micropatterns with high resolution down to 1 µm (Figure 11a). Importantly, the patterned films of silk‐PEDOT blends degraded over several days with the presence of a protease enzyme, allowing the biodegradability for in vivo application. Furthermore, they demonstrate the flexible biosensor devices using patterned PEDOT:PSS and silk protein blend. The patterned PEDOT:PSS showed excellent sensitivity and selectivity of glucose in PBS solutions while fructose, sucrose, and galactose lacked a response (Figure 11b). This work demonstrates a representative example of patterned electroactive organic molecular materials for high‐performance biosensors, especially with enzymatic degradation characteristics.

Cardiac patch is one of the most important treatment methods for myocardial infarctions. In order to achieve online monitoring and reporting of cardiac tissue performance while realizing the scaffold function of the cardiac patch at the same time, Dvir and coworkers developed a microelectronic cardiac patch that integrates cardiac cells with an electroactive polymer of polypyrrole and a 3D nanocomposite scaffold (Figure 11c,d).^[^ ^24^ ^]^ Using microelectronics processing technology, they fabricated a complex, freestanding, porous electronic mesh with multiple electrodes. This mesh‐based microelectronic cardiac patch could record tissue function and provide electrical stimulation to cardiac cells (Figure 11e,f). Specifically, the nanofiber scaffold was designed to support the 3D microenvironment for cardiac tissue growth and tissue assembly. In addition, the mesh‐like structure of hydrogel with negatively charged chondroitin 4‐sulfate enables the encapsulation of positively charged protein via electrostatic interactions and the controlled release through shrinking and neutralization of charge. Thus, PPy was used as an electroactive polymer to promote tissue growth with controlled drug release in the patch microenvironment. The microelectrodes could sense the tissue's electrical activity and provide electrical stimulation to cells and tissues (Figure 11g). Based on these multiple functions, the microelectronic cardiac patch exhibited excellent electronic properties for theragnostic by recording cellular electrical activity and delivering electrical stimulation on demand.

Aside from the organization‐dependent conduction mechanisms present in both biotic and abiotic systems, the inherent contact of the biotic‐abiotic interfaces plays a role in determining the electroactive nature of bioelectronic systems.^[^ ^26^, ^168^, ^169^ ^]^ Indeed, since ion or electron exchange is facilitated at this contact site, optimization of carrier/charge transport in bioelectronic systems has received significant attention over the years.^[^ ^170^ ^]^ The distance between the abiotic and biotic components plays a pivotal role in creating a functional electroactive system, with an optimal distance of less than a few nanometers between the two surface interfaces.^[^ ^171^, ^172^ ^]^ Due to this small optimal distance, current biotic‐abiotic manufacturing techniques can lead to biotic surfaces further from the abiotic substrate left without proper electron exchange. In a recent study to address the shortcomings of current bioelectronic systems reliant on biotic‐abiotic interfaces, Yu et al. improved electron exchange at the biotic‐abiotic interface through the implementation of a conductive layer by directly modifying individual cell membranes.^[^ ^173^ ^]^ McCuskey et al. developed a new bioelectronic composite through the charge transfer from the electroactive bacteria *Shewanella oneidensis* MR‐1 to a self‐doped conjugated polyelectrolyte (CPE‐K).^[^ ^174^ ^]^ Wang et al. designed a high‐conductivity hydrogel as a bioadhesive ultrasoft brain‐machine interface to acquire electroencephalographic signals based on PEDOT nanoparticles hybridized with dopamine methacrylate.^[^ ^175^ ^]^ Wang and coworkers demonstrated wafer‐scale piezoelectric biocompatible films based on *γ*‐glycine and polyvinyl alcohol (PVA), showing significant in vivo piezoelectric response to the muscle stretch.^[^ ^176^ ^]^ Altogether, these works focused on biotic‐abiotic interfacing have promoted a better understanding of charge transport at biological interfaces and the development of electrophysiological devices based on artificial hybrid biosystems.

Although this review focuses on electroactive systems with potential for biomedical applications, it is worth noting that the aforementioned cellular patterning and tissue organization techniques are also applicable to and have been demonstrated for electrogenic bacteria micro‐ and nano‐system design; some examples of which are already mentioned above.^[^ ^177^ ^]^ Electrogenic bacteria are defined by their capability to bidirectionally transfer electrons—being able to both release electrons generated metabolically across the cell membrane and intracellularly intake exogenous electrons. Leveraging the ability of these microbes to serve as either electron donors or acceptors allows them to be patterned and integrated as microbial fuel cells in sensor network design or as biosynthesized nanowires.^[^ ^178^ ^]^ Integrating the ordered structures of electroactive polymers and biomolecules can enhance the interactions between electroactive microorganisms and electrodes for improved extracellular charge transfer,^[^ ^174^, ^177^ ^]^ toward potential applications for microbial biosensors and bioreactors.

## 4 Summary and Perspective

The development of high‐performance electroactive organic molecular materials requires not only careful molecular design and material preparation, but also integration in related devices and applications, whereby the multi‐scale order of materials will play a critical role. Controlling the multi‐scale order of electroactive biotic and organic abiotic systems can enable a systematic modulation of conduction phenomena in physiologically‐relevant environments—from the regulation of intrachain transport in conjugated polymers to supporting the aligned ion‐based signaling across excitable tissues; therefore, benefiting the overall function of the electroactive systems discussed herein. Here, we summarized in detail the recent progress in engineering the multi‐scale organization of electroactive organic molecular materials, including synthetic conjugated molecules and polymers, as well as biomolecule‐based materials. Towards achieving multi‐scale order, we highlight the principles and applications of techniques such as molecular self‐assembly, lithography‐based methods, and printing techniques. From the discussion above, it is clear that the strategies for controlling multi‐scale organization of conducting polymers and biomolecule‐inspired materials, which are also applicable for electroactive living cells and tissues, play a key role in the advancement of bioelectronic technologies. Despite the considerable progress that has been achieved by researchers in the bioelectronics community to date, the following challenges should still be considered and addressed to further catalyze the frontiers of this field:

1) While most materials fabrication approach can be used to impart organization on polymeric or biomolecular materials at multiple length scales, these materials are still often processed with these techniques using organic solvents. Aqueous processability of these materials still needs improvement to enable the inclusion of living cells even during the fabrication steps.
2) Although these current patterning methods are effective in fabricating specific ordered patterns of electroactive organic molecular materials at different length scales, it is still difficult to precisely control the multi‐scale order from nano‐ to macroscale altogether within the same system. However, integration of multiple organization approaches as one fabrication method for electroactive organic materials remains challenging. We believe that an ingenious combination of molecular self‐assembly with one or more micro‐/macroscopic fabrication techniques will facilitate the adaptation of more powerful organization techniques for organic bioelectronic systems in the future.
3) Electrical stimulation and response are common in biological systems, but a seamless integration of organized electroactive organic networks with biological systems still needs further improvement. Transformative improvements on these approaches will enable bioelectronic systems with longer‐term, higher performing capabilities toward what is currently possible for biochemical process monitoring, cell stimulation, tissue engineering, and precision medicine.
4) There is also a critical need for a better understanding of how the structural organization of organic materials evolves upon interfacing with cells. It is not well understood whether the traction or contractile forces exerted by cells on substrates significantly alter microstructural organization of macromolecular substrates, and in turn for electroactive materials, impacting their performance. The impact of deposition of self‐produced extracellular matrix from adherent cells on electrodes/substrates also needs to be better understood.
5) The mechanical properties, such as flexure and rheology, are impacted by the entanglement of chains comprising organic networks. Bridging the structural organization needs of electroactivity and the dynamic requirements for mechanical properties that match the native physiological environments is important for the advancement of implantable bioelectronic organic systems.

**Figure 12:**
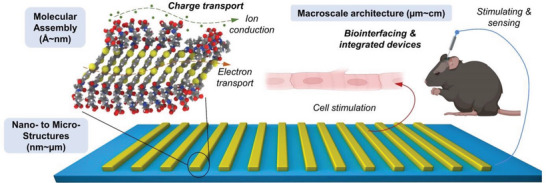
Schematic illustration of the importance of organization of components at various length scales when considering the integration of charge‐transporting organic materials and electroactive cells to create bioelectronic systems.

In summary, we highlight herein the impact of multi‐scale structural organization on regulating the efficiency of facilitating the conduction mechanisms, both electronic and ionic in nature, in electroactive organic systems while interfacing and maintaining a seamless communication between the living and non‐living components of bioelectronic systems (**Figure** **12**). As we push the boundaries of future bioelectronic systems, organization‐dependent conduction mechanisms in both biotic and abiotic components are critical in engineering the optimal performance of these interfaced systems. In summary, achieving precision organization across length scales of electroactive systems will benefit their performance and functionalities for biointerfacing applications. It is our hope that the recent progress summarized in this review, along with the provided insights on pathways for addressing the challenges in engineering the organization of electroactive organic systems, will help push forward the utility of these electroactive systems toward the future of bioelectronics for broader biomedical applications.

## Conflict of Interest

The authors declare no conflict of interest.
